# The survival effect of ovary preservation in early stage endometrial cancer: a single institution retrospective analysis

**DOI:** 10.1186/s13048-020-00698-5

**Published:** 2020-08-22

**Authors:** Wonkyo Shin, Sang-Yoon Park, Sokbom Kang, Myong Cheol Lim, Sang-Soo Seo

**Affiliations:** 1 Center for Gynecologic Cancer, Graduate School of Cancer Science and Policy, National Cancer Center, 323 Ilsan-ro, Ilsandong-gu, Goyang-si, Gyeonggi-do 10408 Republic of Korea; 2 Common Cancer Branch, Research Institute Graduate School of Cancer Science and Policy, National Cancer Center, Goyang, Republic of Korea; 3 Precision Medicine Branch, Graduate School of Cancer Science and Policy, National Cancer Center, Goyang, Republic of Korea; 4 Department of Cancer Control & Population Health, Graduate School of Cancer Science and Policy, National Cancer Center, Goyang, Republic of Korea; 5 Center for Clinical Trials, Graduate School of Cancer Science and Policy, National Cancer Center, Goyang, Republic of Korea; 6 Cancer Healthcare Research Branch, Graduate School of Cancer Science and Policy, National Cancer Center, Goyang, Republic of Korea

**Keywords:** Endometrial cancer, Early stage, Ovary preservation, Menopause

## Abstract

**Purpose:**

We investigated the effect of ovary preserving surgery in early International Federation of Obstetrics and Gynecology (FIGO) stage endometrial cancer patients.

**Methods:**

Medical records were retrospectively reviewed for 539 patients who were diagnosed with early stage endometrial cancer between Jan 2006 and Dec 2017. Patients were categorized into ovary preservation and ovary removal groups. Demographics, recurrence free survival (RFS), and five-year overall survival (OS) rate were compared, and the clinical factors affecting survival were evaluated by univariate and multivariate analysis.

**Results:**

The median follow-up period was 85 months (range, 6–142 months), and the median age was 52.7 years. The mean age was higher in the ovary removal group than in the ovary preservation group (54.4 vs 40.94 years; *P* < 0.001). The ovary preservation group showed an earlier FIGO stage than the ovary removal group (*P* = 0.0264). There was a greater incidence of adjuvant chemotherapy administration in the removal group. There were no statistical differences in other baseline characteristics. When comparing the RFS and OS rates, there were no statistical differences between the preservation and removal groups. (recurrence free rate 98.5% vs 92.7%, *p = 0.4360*, and 5-year survival rate 98.6% vs 93.0%, *p = 0.0892*, respectively). Endometrioid histology (*p = 0.006*) and post-operative adjuvant chemotherapy (*p = 0.0062*) were related to OS, and adjuvant chemotherapy (*p < 0.001*) and radiotherapy (*p = 0.005*) were related to RFS.

**Conclusions:**

Ovary preservation in early stage endometrial cancer is worth considering, as it does not affect survival in early stage endometrial cancer patients.

## Background

Endometrial cancer has the highest incidence in gynecological cancers in Western countries [1], and the incidence is also increasing in Korea [2]. Conversely, endometrial cancer has a higher rate of early diagnosis than other gynecologic and solid cancers because symptoms such as irregular bleeding or discharge are easily detected by patients and diagnosis is possible with a simple endometrial curettage or hysteroscopic endometrial biopsy [3].

According to the National Comprehensive Cancer Network (NCCN) guidelines, early stage endometrial cancer is treated with total hysterectomy, bilateral salpingo-oophorectomy, and lymph node dissection, with subsequent staging according to the pathologic report. Adjunctive total salpingo-oophorectomy is the standard treatment option, because of the possibility of occult tumor cells in the ovary and the fact that endometrial cancer is advanced by ovarian hormones [4, 5]. The incidence of ovarian tumors in patients with endometrial cancer is as 7% [6]. However, surgical menopause caused by removing the ovaries can induce other complications, such as hot flushes, night sweats, vaginal dryness, insomnia, osteoporosis, cardiovascular problem, sexual dysfunction, and cognitive problems that can affect survival and quality of life [7–11]. Exogenous hormone replacement therapy may relieve these menopausal symptoms but can also induce other complications [12, 13].

Meta-analysis [14] and other research [4, 5, 15, 16] on ovarian preservation surgery in young, premenopausal early stage endometrial cancer patients has produced conflicting results. Some studies claim that it is safer to remove the ovaries, although preservation of the ovary generally does not affect patient recurrence or survival. However, in some meta-analyses and reviews, much of the patient data (34.9%) is too old records [4], therefore, there is a risk of inaccurate medical records. Further, studies included incidentally diagnosed cancer patients who were regarded as having benign disease such as leiomyoma or adenomyosis prior to surgery, and only had hysterectomy planned without salpingo-oophorectomy.

In addition, many patients receive adjuvant treatment such as radiotherapy or chemotherapy after ovarian preserving surgery, which can further damage ovarian functions. These cases do not represent true ovarian preservation or help to reduce the complications of surgical menopause.

This retrospective study compares the survival of early International Federation of Obstetrics and Gynecology (FIGO) stage endometrial cancer patients who have the ovaries preserved with those who had the ovaries removed in a real clinical setting.

## Methods

**Fig. 1:**
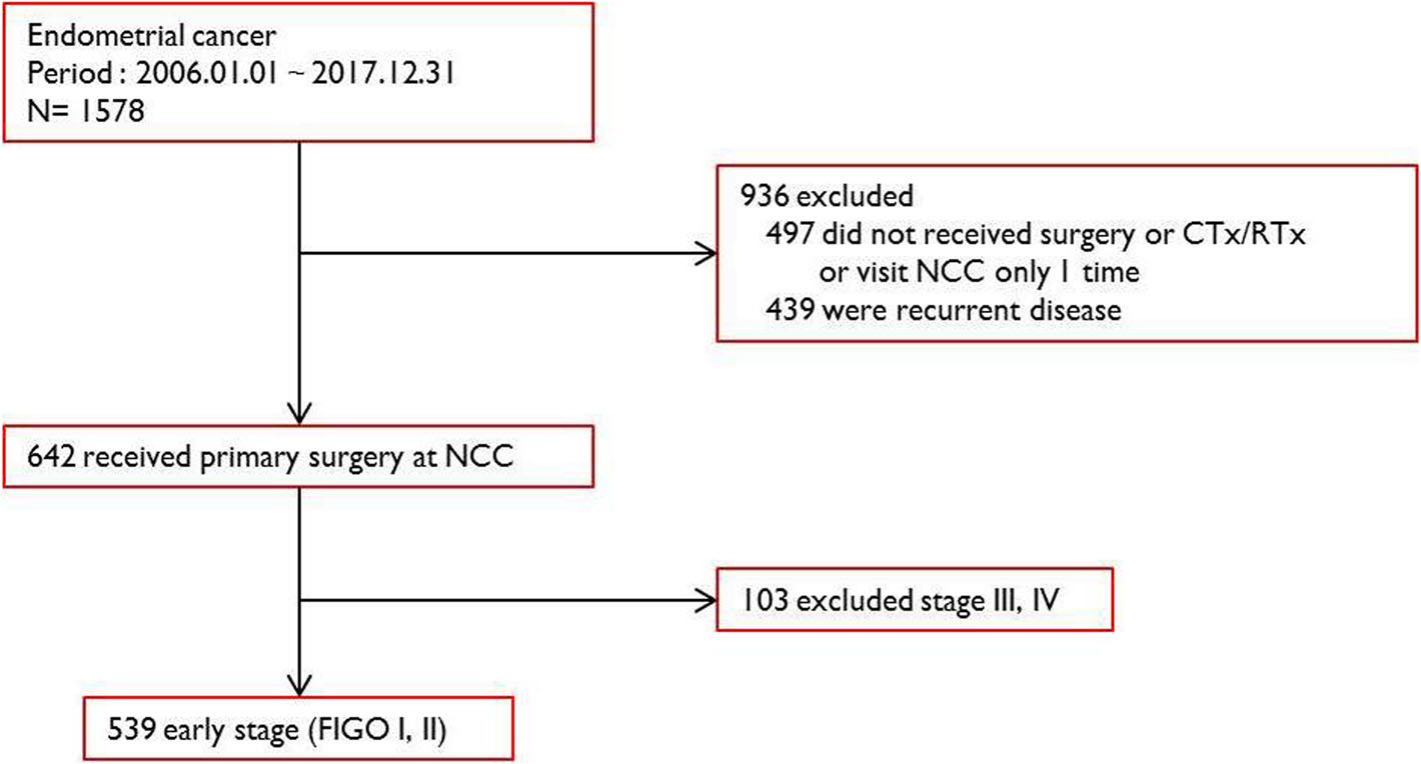
Study flow chart

Medical records of patients with endometrial cancer who were newly diagnosed by endometrial biopsy and treated at National Cancer Center in South Korea between January 2006 and December 2017 were reviewed. A total of 1578 endometrial cancer patients visited our outpatient clinics; however, 497 patients visited only once for counseling or a second opinion, and 439 patients had recurrent disease. Another 103 patients were excluded due to advanced cancer status (FIGO stage III or IV), which resulted in 539 patients who had been diagnosed and treated in our center for early (FIGO stage I or II) endometrial cancer (Fig. 1). Clinical factors including age at diagnosis, FIGO stage, FIGO grade, histology of the surgically removed tissues, surgical approach method, radicality of hysterectomy, lymph node dissection, and adjuvant chemotherapy and radiotherapy were collected.

Correlations of variables were assessed using the Fisher’s exact or Student *t-*test. Five year overall survival (OS) rates and recurrence free survival (RFS) rates were estimated by Kaplan-Meier analysis. The log-rank test was used to compare survival curves. Cox regression analysis was performed to determine the predictive factors for prognosis with hazard ratios (HRs). *P* values <.05 were considered to be significant. This retrospective study was approved by the institutional review board of our institution (IRB No. NCC2019–0272).

## Results

**Fig. 2:**
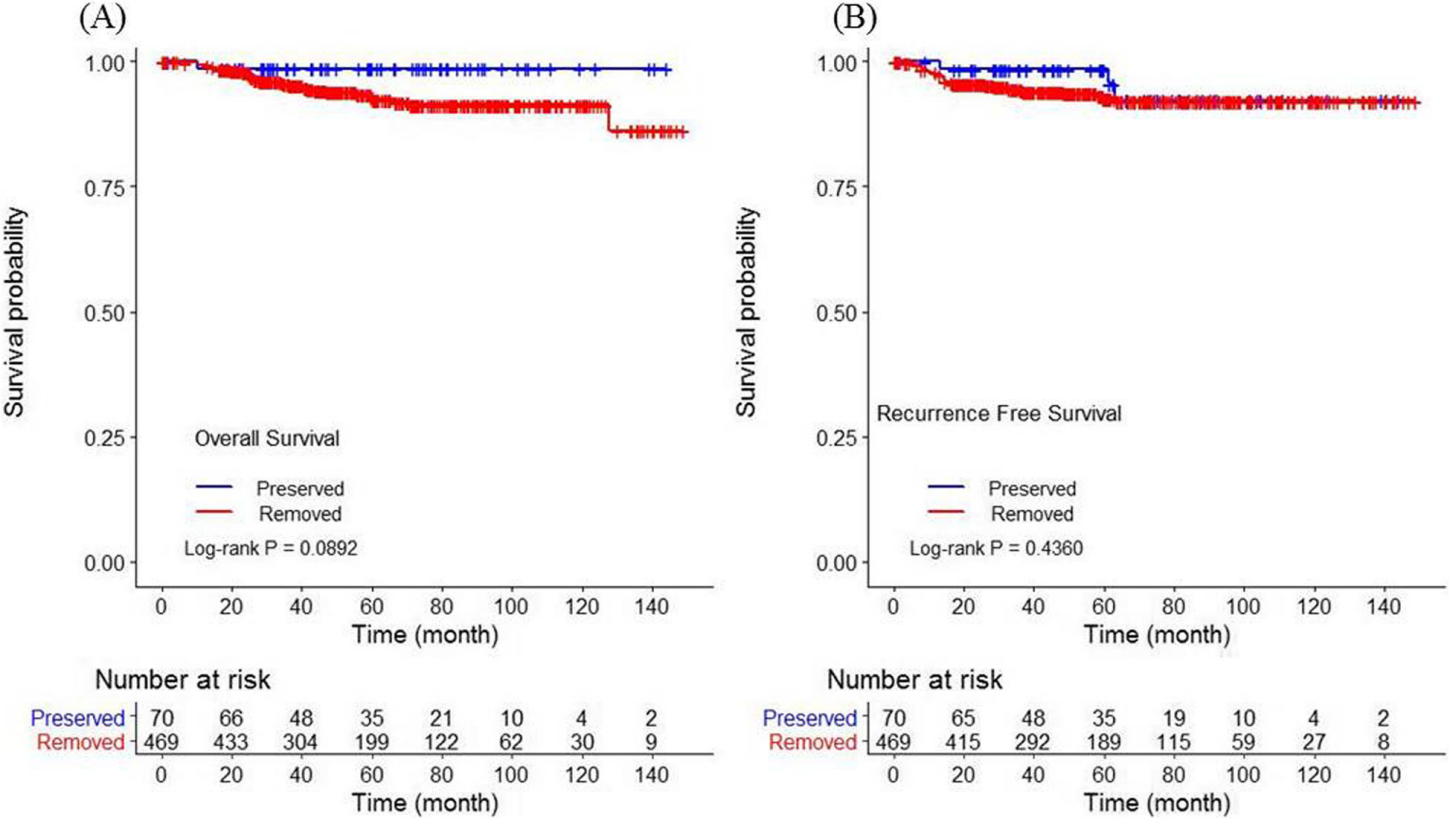
Overall survival and recurrence free survival

The 539 patients included 469 in the ovarian removal group and 70 in the ovarian preservation group. The ovarian removal group was significantly older than the ovarian preservation group (*P* < 0.001). The FIGO stage was earlier in the ovarian preservation group (*P* = 0.0264). Lymph node dissection was more frequently performed in the ovarian removal group. There was no significant difference between the two groups in terms of surgical approach method, radicality of hysterectomy, or the administration of adjuvant chemotherapy or radiotherapy (Table 1). The five-year OS) and RFS) graphs showed no significant differences between ovarian preservation and removal groups (OS: 98.6% vs. 93.0%, *P* = 0.0892, and RFS: 98.5% vs 92.7%, *P* = 0.436, respectively) (Fig. 2). Adjusted univariate analysis was performed for FIGO stage and patient age. Univariate analysis demonstrated that FIGO grade, histology, and adjuvant chemotherapy were significantly related to RFS and OS. Adjuvant radiotherapy was related to RFS only. Pelvic lymph node dissection was related to OS only. Neither surgical approach nor radicality of hysterectomy were related to RFS or OS. In multivariate analysis, histology and adjuvant chemotherapy were related to OS, and adjuvant chemotherapy and radiotherapy were related to RFS (Table 2). Compared to endometrioid histology, non-endometrioid histology showed relatively low RFS and OS. Ovarian preservation or removal was not related to RFS or OS.

**Table 1:** Patient baseline characteristics

Variables	Total	Ovary Preserved	Ovary Removed	
	***N*** = 539	***N*** = 70	***N*** = 469	***P***-value
FIGO Stage				
IA	390 (72.36)	60 (85.71)	330 (70.36)	0.0264
IB	126 (23.38)	8 (11.43)	118 (25.16)	
II	23 (4.27)	2 (2.86)	21 (4.48)	
FIGO grade				
1	293 (54.36)	41 (58.57)	252 (53.73)	0.289
2	141 (26.16)	21 (30)	120 (25.59)	
3	52 (9.65)	5 (7.14)	47 (10.02)	
Etc.	53 (9.83)	3 (4.29)	50 (10.66)	
Histology				
Endometrioid	461 (85.53)	66 (94.29)	395 (84.22)	0.2329
Serous	31 (5.75)	2 (2.86)	29 (6.18)	
CCC	20 (3.71)	1 (1.43)	19 (4.05)	
Mucinous/mixed/Undifferentiated/NE/etc.	27 (5.01)	1 (1.43)	26 (5.54)	
Age (year)				
mean ± SD	52.68 ± 10.42	40.94 ± 9.56	54.43 ± 9.36	<.0001
< 30	11 (2.04)	8 (11.43)	3 (0.64)	<.0001
31 ~ 35	19 (3.53)	13 (18.57)	6 (1.28)	
36 ~ 40	39 (7.24)	21 (30)	18 (3.84)	
40 ~ 45	51 (9.46)	11 (15.71)	40 (8.53)	
45>	419 (77.74)	17 (24.29)	402 (85.71)	
Approach				
Laparoscopy	395 (73.28)	60 (85.71)	335 (71.43)	0.0366
Laparotomy	138 (25.6)	10 (14.29)	128 (27.29)	
Etc.	6 (1.11)	0 (0)	6 (1.28)	
Hysterectomy - radicality				
A	489 (90.72)	63 (90)	426 (90.83)	0.7491
B	18 (3.34)	2 (2.86)	16 (3.41)	
C	27 (5.01)	5 (7.14)	22 (4.69)	
Not done	5 (0.93)	0 (0)	5 (1.07)	
PLND				
No	112 (20.78)	23 (32.86)	89 (18.98)	0.0076
Yes	427 (79.22)	47 (67.14)	380 (81.02)	
PALND				
No	251 (46.57)	51 (72.86)	200 (42.64)	<.0001
Yes	288 (53.43)	19 (27.14)	269 (57.36)	
Adjuvant Chemotherapy				
No	455 (84.42)	66 (94.29)	389 (82.94)	0.0147
Yes	84 (15.58)	4 (5.71)	80 (17.06)	
Adjuvant radiotherapy				
No	468 (86.83)	64 (91.43)	404 (86.14)	0.2224
Yes	71 (13.17)	6 (8.57)	65 (13.86)	

*CCC* clear cell carcinoma, *NE* neuroendocrine tumor, *PLND* pelvic lymph node dissection, *PALND* para-aortic lymph node dissection

**Table 2:** Univariate and multivariate analysis of prognostic factors

variables	Overall survival				Recurrence Free survival			
	Adjusted model^**a**^		Multivariable^**b**^		Adjusted model^**a**^		Multivariable^**b**^	
	HR(95% CI)		HR(95% CI)		HR(95% CI)		HR(95% CI)	
	*N* = 539/EVNET = 31	*p*-value	*N* = 539/EVNET = 31	*p*-value	*N* = 539/EVNET = 33	*p*-value	*N* = 539/EVNET = 33	*p*-value
Ovary								
preserved	1(ref)				1(ref)			
removed	2.058 (0.266–15.915)	0.4892			0.865 (0.245–3.052)	0.8215		
FIGO grade								
1	1(ref)	**0.0103**			1(ref)	**0.0339**		
2	2.305 (0.869–6.116)	0.0935			2.418 (1.052–5.556)	0.0376		
3	4.215 (1.324–13.42)	0.0149			1.176 (0.255–5.416)	0.8355		
etc	5.102 (1.838–14.162)	0.0018			3.807 (1.465–9.894)	0.0061		
Histology								
Endometrioid	1(ref)	**0.0013**	1(ref)	**0.0006**	1(ref)	**0.0322**		
Serous	5.333 (2.206–12.892)	0.0002	5.969 (2.437–14.617)	<.0001	3.548 (1.389–9.062)	0.0081		
CCC	3.381 (0.968–11.806)	0.0562	3.729 (1.064–13.072)	0.0397	3.031 (0.894–10.274)	0.075		
Mucinous/mixed/Undifferentiated/NE/etc	3.212 (0.921–11.196)	0.067	3.373 (0.963–11.808)	0.0572	1.609 (0.374–6.918)	0.5224		
approach								
Laparoscopy	1(ref)	**0.027**			1(ref)	0.4568		
Laparotomy	2.298 (1.058–4.992)	0.0355			1.604 (0.765–3.36)	0.2106		
etc	7.88 (0.974–63.726)	0.0529			–	0.9883		
Hysterectomy - radicality								
A	1(ref)	0.2591			1(ref)	0.9624		
B	–	0.9901			0.598 (0.073–4.913)	0.6322		
C	0.443 (0.091–2.159)	0.3139			0.776 (0.162–3.704)	0.7503		
Not done	6.183 (0.769–49.733)	0.0868			–	0.9887		
PLND								
No	1(ref)				1(ref)			
Yes	0.371 (0.162–0.852)	**0.0194**			0.549 (0.24–1.256)	0.1556		
PALND								
No	1(ref)				1(ref)			
Yes	0.848 (0.407–1.766)	0.6593			1.008 (0.491–2.068)	0.9826		
Adjuvant chemotherapy								
No	1(ref)		1(ref)		1(ref)		1(ref)	
Yes	2.083 (0.969–4.477)	**0.0602**	0.318 (0.137–0.736)	**0.0074**	2.864 (1.396–5.873)	**0.0041**	3.414 (1.649–7.069)	**0.0009**
Adjuvant radiotherapy								
No	1(ref)				1(ref)		1(ref)	
Yes	0.659 (0.249–1.744)	0.4006			2.655 (1.163–6.059)	**0.0204**	3.256 (1.428–7.423)	**0.005**

*CCC* clear cell carcinoma, *NE* neuroendocrine tumor, *PLND* pelvic lymph-node dissection, *PALND* para-aortic lymph node dissection

^a^ Adjusted model: adjuted age at diagnosis (≤50 vs > 50), FIGO stage

^b^ Multivariableb: adjuted age at diagnosis (≤50 vs > 50), FIGO stage

## Discussion

There is no clear consensus on the surgery scale in early stage endometrial cancer patients. Traditionally, total hysterectomy, bilateral salpingo-oophorectomy, and lymph node dissection and omentectomy for staging have been performed. According to the NCCN; American Society of Clinical Oncology (ASCO); and European Society for Medical Oncology, European Society of Gynaecological Oncology, and European Society for Radiotherapy & Oncology (ESMO-ESGO-ESTRO) guidelines [3, 17, 18], ovarian preservation can be considered in pre-menopausal early stage endometrial cancer patients. While oophorectomy removes any occult ovarian metastatic tumor and reduces the ovarian cancer risk, surgical menopausal problems can result.

Previous studies have shown that preserving the ovary does not affect survival in patients with early stage endometrial cancer who are not menopausal, compared with those who have had the ovaries removed [4, 5, 14, 15]. Previous studies are compared in Table 3. These retrospective studies were conducted in the United States, China, and Korea, conducted mainly with pre-menopausal women as patient groups. Prospective research has been difficult to perform as recruitment of early stage and premenopausal patients has been challenging. Their results showed that in the case of early stage and low FIGO grade, preservation of the ovary did not affect the prognosis of the patient. Recently, a systemic review summarized previous studies, with more than 10,000 cases, and revealed an increase in OS and no shortening of RFS. In the early stages of premenopausal women, ovarian preservation may be a viable treatment option [19]. The results are similar to ours. However, as previously noted, this research has several limitations, including a large portion of patients with benign disease, the inclusion of patients with adjuvant chemotherapy or radiotherapy after preserving the ovaries [5, 16], or including patients with old records [4]. There are many reports of ovarian function deterioration after radiation or chemotherapy in pre-menopausal women [20–23]. These cases demonstrate that preserving the ovaries does not result in maintaining ovarian function, since adjuvant treatment can also induce menopause.

**Table 3:** Previous studies about ovarian preservation in endometrial cancer patients

Study	Year	Country	Period	Patients	Age	Stage	Grade	Histology	Follow up period (month)	Survival benefits
Wright	2009	USA	1988–2004	402/2867	45	Ia 64%, Ib 33%, Ic 3%	I 79%, II 14%, III 3%	endo	1–121	NS
Lee	2009	Korea	1993–2005	123/−	45	Ia 74%, Ib 23%, Ic 3%	I 70%, II 17%, III 2%	Endo (86%)	1–125	No recur, 5-yr OS:98%
Sun	2012	China	2002–2010	34/132	45	Ia 71%, Ib 22%, Ic 7%	I 70%, II 20%, III 10%	Endo (97%)	27–122	NS
Lee	2013	Korea	1997–2008	176/319		Ia 89% Ib 5% II 6%	I 78% II 18% III 4%	endo	6–208	NS
Wright	2016	USA	1998–2012	1121/14527	< 50	Ia 89% Ib 8% II 3%	I 60% II 27% III 6%	endo	61, median	NS
Wang	2016	China	2009–2015	25/76	< 45	Ia 87%, Ib 13%	I 75%, II 21%, III 40%	Endo (99%)		NS

*NS* non-specific

Endometrial cancer in premenopausal women has been shown to be hormone related, have early stage, no myometrial invasion, and good prognosis [24]. If metastatic or synchronous malignancy has not been found in the ovary during surgery, ovarian preservation may be performed; therefore, surgical menopause of the patient is not induced, which may be more beneficial to women’s health. We recommend to consider the pathology of the tumor and the necessity of maintaining fertility before surgery.

Although this retrospective study has some limitations, this is the first study in which all patients had been diagnosed with endometrial cancer through preoperative endometrial biopsy, and had ovarian preservation or removal planned in advance of surgery, after confirming no other distant metastasis through computed tomography scan and lab test. Further, a relatively low ratio of patients was administered adjuvant treatment, so this study may serve as a reliable reference for early FIGO stage endometrial cancer. However, this study has limited data about the side effects of each group, especially menopausal problems that occur in the ovarian removal group. Long-term follow-up of adverse effects in postoperative patients may reveal significant differences in patients who have undergone surgical menopause. Second, although a small number of patients received adjuvant treatment after surgery, that treatment was related to survival; consequently, a more accurate group selection process is warranted for future studies.

Cancer survival and life expectancy after diagnosis are increasing, and quality of life issues are becoming more important. It is time forFurther prospective research to confirm whether it is more favorable to remove the ovaries to decrease risk of recurrence or to maintain patient quality of life through ovarian preservation.

## Conclusions

Ovarian preserving surgery in early stage endometrial cancer is a beneficial option for premenopausal patients and is not related to disease recurrence or overall survival rate. More precise stratification analysis is needed to determine which additional groups may safely preserve the ovary.

## Data Availability

All data generated or analysedanalyzed during this study are included in this published article.

## Abbreviations

ASCO: American Society of Clinical Oncology
ESMO-ESGO-ESTRO: European Society for Medical Oncology, European Society of Gynaecological Oncology, and European Society for Radiotherapy & Oncology
FIGO: International Federation of Obstetrics and Gynecology
HRs: Hazard ratios
IRB: Institutional review board of our institution
NCCN: National Comprehensive Cancer Network
RFS: Recurrence free survival
OS: Overall survival
